# Mung Bean (*Vigna radiata* L.): Bioactive Polyphenols, Polysaccharides, Peptides, and Health Benefits

**DOI:** 10.3390/nu11061238

**Published:** 2019-05-31

**Authors:** Dianzhi Hou, Laraib Yousaf, Yong Xue, Jinrong Hu, Jihong Wu, Xiaosong Hu, Naihong Feng, Qun Shen

**Affiliations:** 1 Key Laboratory of Plant Protein and Grain Processing, National Engineering and Technology Research Center for Fruits and Vegetables, College of Food Science and Nutritional Engineering, China Agricultural University, Beijing 100083, China; xiaozhihou90@126.com (D.H.); laraibyousaf786@hotmail.com (L.Y.); xueyong@cau.edu.cn (Y.X.); hujr@cau.edu.cn (J.H.); jihong-wu7268@163.com (J.W.); huxiaos@263.net (X.H.); 2 Institute of Economic Crops, Shanxi Academy of Agricultural Sciences, Fenyang 032200, China; fengnh@126.com

**Keywords:** mung bean, bioactive compounds, polyphenols, polysaccharides, peptides, health benefits

## Abstract

Mung bean (*Vigna radiata* L.) is an important pulse consumed all over the world, especially in Asian countries, and has a long history of usage as traditional medicine. It has been known to be an excellent source of protein, dietary fiber, minerals, vitamins, and significant amounts of bioactive compounds, including polyphenols, polysaccharides, and peptides, therefore, becoming a popular functional food in promoting good health. The mung bean has been documented to ameliorate hyperglycemia, hyperlipemia, and hypertension, and prevent cancer and melanogenesis, as well as possess hepatoprotective and immunomodulatory activities. These health benefits derive primarily from the concentration and properties of those active compounds present in the mung bean. Vitexin and isovitexin are identified as the major polyphenols, and peptides containing hydrophobic amino acid residues with small molecular weight show higher bioactivity in the mung bean. Considering the recent surge in interest in the use of grain legumes, we hope this review will provide a blueprint to better utilize the mung bean in food products to improve human nutrition and further encourage advancement in this field.

## 1. Introduction

Growing clinical evidence suggested that consumption of calorie-rich diets, that are high in fat and carbohydrate but low in protein, has led to increased rates of metabolic syndromes, such as hyperglycemia, dyslipidemia, and inflammation [1]. A variety of plant-based functional foods have been recommended by many worldwide health organizations, prompting a call for serious changes in dietary patterns, in order to improve health statuses and prevent chronic diseases [2,3]. Legumes (Fabaceae/Leguminosae) are considered the second most important human food crops, just after the cereals (Gramineae). However, legume seeds constitute an essential part of the human diet as they are excellent sources of proteins, bioactive compounds, minerals, and vitamins, in comparison with cereals, and are referred to as “the poor man’s meat” [4,5].

The mung bean (*Vigna radiata* L.) is one of the most important edible legume crops, grown on more than 6 million ha worldwide (about 8.5% of the global pulse area) and consumed by most households in Asia. Due to its characteristics of relatively drought-tolerant, low-input crop, and short growth cycle (70 days or so), the mung bean is widely cultivated in many Asian countries (concentrated mainly in China, India, Bangladesh, Pakistan, and some Southeast Asian countries) as well as in dry regions of southern Europe and warmer parts of Canada and the United States [6]. In the predominantly cereal-based diets of China and India, the mung bean has been consumed as a common food for centuries. The mung bean contains balanced nutrients, including protein, dietary fiber, minerals, vitamins, and significant amounts of bioactive compounds [7]. For those individuals who cannot afford animal proteins or those who are vegetarian, the mung bean is of a comparatively low-cost and has a good source of protein for them. Furthermore, mung bean protein is easily digestible, as compared to protein in other legumes [8,9]. Consumption of the mung bean combined with cereals has been recommended to significantly increase the quality of protein, because cereals are rich in sulfur-containing amino acids but deficient in lysine [10]. A 3:4 ration of mung bean protein with rice protein, obtaining the highest chemical amino acid score (72), was suggested as good for consumption [6]. It was found that the protein digestibility of the rice-mung bean combination diet was 84.4% of that observed for the rice-meat combination diet in infants, which can almost meet human needs for protein [11]. Moreover, the plant-source proteins could help to reduce the land occupation and greenhouse gas emissions as compared to the animal-source proteins, achieving a better compromise between dietary habits and environmental protection [12]. The mung bean induces less flatulence and is well tolerated by children [13]. In many studies, the mung bean was recommended as a supplement for preparing an infant’s weaning food because of its high protein content and hypoallergic properties [14,15]. In Pakistan, approximately 25% of all iron in the diet is provided by pulses, and the mung bean is consumed by all households [16]. Despite this, the presence of anti-nutritional factors in the mung bean may limit the biological value of its nutrients. For example, phytic acid can bind to several important divalent cations such as iron, zinc, calcium, and magnesium. The insoluble complexes formed in result of this binding can limit the mineral absorption and utilization in the small intestine [17]. However, the anti-nutritional factor can be reduced or eliminated by using various processing methods, such as fermentation, germination, dehulling, and cooking [18,19]. After germination, the phytic acid contents declined in mung beans by 76%, and bioavailability values of zinc and iron increased were 3.0 and 2.4 times higher than that of raw mung beans, respectively [20]. Therefore, the antinutritional properties do not hinder the use of the mung bean.

In addition to the nutritional properties of the mung bean, the Compendium of Materia Medica (the “*Bencao Gangmu*”), a well-known Chinese pharmacopoeia, has recorded that it can be utilized as Chinese traditional medicine for its detoxification activities, recuperation of mentality, ability to alleviate heat stroke, and regulation of a gastrointestinal upset. Interestingly, apart from the ancient description, recent studies have identified many other potential health benefits of the mung bean, such as its hypoglycemic and hypolipidemic effects and its antihypertensive, anticancer, anti-melanogenesis, hepatoprotective, and immunomodulatory properties beyond meeting basic nutrient requirements [21,22,23,24,25,26]. In support of these health benefits, a considerable number of studies have been conducted to confirm its chemical constituents, especially the polyphenolics, polysaccharides, and peptides [27,28,29].

To our knowledge, a detailed compilation of research regarding the bioactive polyphenols, polysaccharides, and peptides in the mung bean is lacking. Thus, it is necessary to review the studies to provide some reference for experts and scholars. Moreover, the present paper aims to show the potential action mechanisms of the mung bean on its disease prevention and health improvement to promote its consumption as an alternative function food in Asian countries and other countries. Limitations and recommended future directions of study on the mung bean are also discussed to encourage advancement in the field.

## 2. Polyphenolics

**Figure 1:**
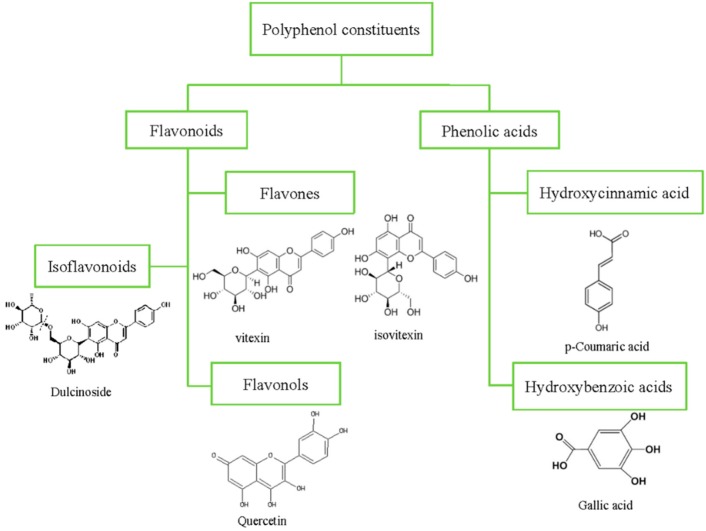
Chemical structures of the main polyphenol constituents in the mung bean.

The mung bean is rich in polyphenolics. The major phenolic constituents in the mung bean are phenolic acids (1.81–5.97 mg rutin equivalent/g), flavonoids (1.49–1.78 mg catechin equivalent/g), and tannins (1.00–5.75 mg/g) [30,31,32,33]. The chemical structures of the main polyphenol constituents in the mung bean are shown in Figure 1. Both the cotyledons and seed coats of mung beans contain phenolics, but most are concentrated in the seed coats. In addition, the composition and content of bioactive compounds in the mung bean depend on many factors, e.g., their cultivars, the color of their seed coats, the climatic and agronomic conditions of their growth, and the extraction and analytical methods [34]. The polyphenol composition and content in the mung bean are shown in Table 1.

As the main secondary metabolite in plants, phenolic acids can be divided into hydroxycinnamic acids and hydroxybenzoic acids, according to their chemical structure. To date, five hydroxycinnamic acids and three hydroxybenzoic acids have been identified in the mung bean. Caffeic, p-coumaric, and t-ferulic acids are the most common hydroxycinnamic acids in the mung bean, while gallic acid is the most frequently occurring hydroxybenzoic acid. In addition, phenolic acids are mainly present in free or bound forms in plant cells. Bound phenolics are considered to exert a greater effect on health benefits, because they are more likely to escape from upper gastrointestinal digestion, along with cell wall materials, and are absorbed into blood plasma through microflora digestion activity [40,41]. Yao et al. found that, among the phenolic acids in the mung bean, ferulic, caffeic, chlorogenic, syringic, p-coumaric, and gentisic acids exist in bound forms [35].

Flavonoids are the most abundant secondary metabolites in the mung bean. Five subclasses of flavonoids, i.e., flavones, flavonols, isoflavonoids, flavanols, and anthocyanins, were found in the mung bean (Table 1). Normally, they accumulate in the plant tissues as conjugates in the forms of glycosylation or esterification, but occasionally they can also be found as aglycones [42]. Flavones (vitexin, isovitexin, isovitexin-6″-*O*-α-l-glucoside, and luteolin) and flavonols (quercetin, myricetin, and kaempferol) are the most abundant flavonoids detected in the mung bean. Vitexin and isovitexin were proved to be the two major flavonoids in the mung bean seed; their contents in the seed coat contributed to 95.6% and 96.8% of the total vitexin and isovitexin, respectively [43,44]. Quantitative analysis demonstrated that the contents of vitexin and isovitexin in the mung bean seed coat were as high as 37.43 mg/g and 47.18 mg/g, respectively [45]. Moreover, these two major flavonoids were also derived and identified from the mung bean broth, a traditional health food in China [46]. Furthermore, Bai et al. studied the plasma pharmacokinetics, bioavailability, and tissue distribution of three flavones (vitexin, isovitexin, and isovitexin-6″-*O*-α-l-glucoside) and one iso-flavonoid (dulcinoside) in rats [39]. The results showed that vitexin (4.58 mg/kg), isovitexin (10.64 mg/kg), isovitexin-6″-*O*-α-l-glucoside (3.40 mg/kg), and dulcinoside (0.26 mg/kg) could be rapidly absorbed and distributed in the heart, spleen, liver, lung, intestine, stomach, kidney, and brain after oral administration (2 g/kg, 95% ethanolic extract of mung bean seeds), and all of them achieved a maximum concentration at around 1.5 h. The concentrations of the three flavones and one iso-flavonoid in all of the tested tissues decreased significantly within 4 h, indicating that they don’t have a trend of long-term accumulation. This may be related to a possible homeostatic relationship between the antioxidant and oxidative status of the serum. In addition, according to the studies of Soccio et al., this was also greatly affected by the analytical methods, and the antioxidant/oxidant balance may provide more integrated and truthful results [47,48]. In addition, the bio-availabilities of vitexin, isovitexin, isovitexin-6″-*O*-α-l-glucoside, and dulcinoside were 5.82, 5.53, 1.37, and 17.07%, respectively. The different types of glycosidic bonds (α or β) and glycosylation sites (C-8 or C-6) in their structure were considered responsible for the significant difference in pharmacokinetic characteristics and bio-availabilities. Interestingly, the four flavones could transfer across the blood-brain barrier. Anthocyanin is an important type of flavonoid in pigmented pulses [49]. It was surprising that this anthocyanin was only detected in the black mung bean, and not in the green mung bean [35].

Analytical studies have shown that germination and fermentation can significantly improve the metabolites in the mung bean [17,50]. After germination, the total contents of phenolic acids and flavonoids, including vitexin and isovitexin, in the mung bean sprouts were significantly increased, up to 4.5 and 6.8 times higher than that of raw mung bean seeds, respectively [38,51]. The fermented mung bean was also found to be a good source of γ-aminobutyric acid. The concentration of γ-aminobutyric acid in the fermented mung bean increased by 7.6-fold to 0.122 g/100 g, while its content in the non-fermented mung bean was 0.016 g/100 g [52,53].

## 3. Polysaccharides

Polysaccharides are proved to play an important role in physiological activities [54]. In recent years, bioactive polysaccharides obtained from the mung bean have attracted increasing attention, and some advances have been made to characterize these polysaccharides. Table 2 shows several polysaccharides isolated from the mung bean and their bioactivities. Most of the reported polysaccharides were prepared from water extracts (solvent-free) of mung beans, and they exhibited antioxidant and immunoregulatory activities [55,56,57]. However, ethylenediaminetetraacetic acid and alkali-soluble polysaccharides isolated from the mung bean were also shown to activate macrophages [28,58]. Ketha et al. investigated the structural properties of an acidic arabinogalactan (AGP-2), isolated from the mung bean Hemicellulose-B, which was proved to be the most potent in the immunomodulation [59]. The structural characterization of the AGP-2 indicated it as a Type II arabinogalactan backbone, composed of a 1, 3, and 1, 3, 6-Galp. The side chains originating at position 6 of the backbone are composed of 1, 6-Galp and terminal arabinose residues. There was a close relationship between the structure and activity of polysaccharides [60]. Thus, structures of polysaccharides remain to be further determined to elucidate the structure-activity relationship.

## 4. Peptides

In addition to serving as dietary nutrients for humans, the proteins of pulses, including the mung bean, also contain amino acid sequences that, upon digestion, release peptides, which may exhibit a certain bioactivity. Additional bioactive properties, such as functioning as angiotensin I-converting enzyme (ACE) inhibitors, antioxidants, and an anticancer asiatic acid carrier, can be provided by these peptides, obtained from the mung bean protein hydrolysate [25,61]. Current studies on the bioactivity of peptides mainly focused on the ACE inhibition effect. The un-hydrolyzed mung bean protein isolates showed no inhibitory activity on ACEs [62]. The activity of mung bean protein peptides was affected by many factors, such as types of hydrolases, enzymatic hydrolysis time, and amino acid compositions, sequences, and molecular weight. The peptides derived from alcalase exhibited the highest degree of hydrolysis and a trichloroacetic acid–nitrogen soluble index, as compared to other enzyme hydrolysates, including neutrase, papain, and protamex [25]. These peptides obtained with alcalase (20 μL/g enzyme/bean protein) for 2 h of hydrolysis time at 55 °C and pH 8.0 were proved to show the highest ACE inhibitory activity with the IC50 value of 0.64 mg protein/mL [62].

Moreover, it was found that three kinds of novel ACE inhibitory peptides isolated from the alcalase hydrolysate of the mung bean protein isolate exerted high ACE inhibitory activity, and their amino acid sequences were identified to be Lys-Asp-Tyr-Arg-Leu, Val-Thr-Pro-Ala-Leu-Arg, and Lys-Leu-Pro-Ala-Gly-Thr-Leu-Phe [63]. The structure-activity correlations among different peptide inhibitors of ACEs indicated that those peptides containing hydrophobic amino acid residues, especially the aromatic amino acids, at each of the three C-terminal positions, appeared to be preferred by ACE as the substrate or competitive inhibitors. Xie et al. suggested that the peptide (<3 kDa), with small molecular weight isolated from thr mung bean protein hydrolysates, showed higher ACE inhibitory and antioxidant activities, including DPPH radical scavenging activity, hydroxyl radical scavenging ability, and metal-chelating activity, than another two peptides, whose molecular weights were 3–10 kDa and >10 kDa, separately [25]. The relatively high concentrations of aromatic amino acids (10.56%) in the amino acid composition of the small molecular weight peptide may be another important explanation for its high activity. Thus, more work is needed to establish the molecular weight profile and amino acid sequence of peptides obtained from mung bean protein hydrolysates, as well as their role in optimizing applications to benefit human health.

## 5. Health Benefits

The mung bean has been consumed as a diet worldwide and plays a vital role in human nutrition, especially as a good source of protein (20.97–32.6%) and active compounds. The mung bean protein has been identified as an effectively excellent source of amino acids, and the essential amino acids in particular, in which many kinds of cereals are deficient [16]. It can be served as a staple food in India and Pakistan, called “dhal”, which meets people’s daily needs for protein and provides sufficient bioavailability. The rich nutrients of the mung bean, such as minerals, iron, dietary fiber, and significant amounts of bioactive phytochemicals also make it a good alternative function food. Furthermore, the polyphenols, polysaccharides, and polypeptides contained in the mung bean all exert antioxidant activity, which can contribute to disease prevention [23,25]. To date, the mung bean and its extracts have shown excellent health implications, such as hypoglycemic and hypolipidemic effects and antihypertensive, anticancer, anti-melanogenesis, hepatoprotective, and immunomodulatory activities (Table 3 and Table 4). The detailed information on the recent reports including in vitro and animal studies is summarized in Table 3 and Table 4.

### 5.1. Hypoglycemic Properties

Generally, besides the genetic pre-dispositions and obesity, long-term high consumption of foods with a high glycemic index is an important influencing factor for triggering diabetes. Several studies indicated that consumption of low glycemic index food is useful in the reduction of diabetes mellitus and obesity [98,99]. In respect of that, mung bean starch has the unique characteristics of having a low glycemic index. Lerer–Metzger et al. reported that a test meal of mung bean starch induced lower glycemic responses than the same amount of wheat starch in normal rats [100]. Furthermore, the digestibility of starch is influenced by the ratio of amylose-amylopectin, thus impacting the postprandial glucose response [101,102]. Mung bean starch contains a higher level of amylose than that of other pulses [103]. Bruzzo et al. evaluated the effects of the chronic consumption of mung bean starch (32% amylose) and cornstarch (0.5% amylose) on a glucose metabolism in normal and diabetic rats [104]. The results indicated that rats fed with the high amylose content of mung bean starch showed a lower glycemic index in comparison with rats fed with waxy cornstarch with low content amylose. This mung bean starch diet was found to be able to increase glucose oxidation in diabetic rats, indicating enhanced peripheral insulin action as well as glucose utilization. A low glycemic index diet is beneficial in normalizing the diet-insulin responses by improving adipocyte insulin-mediated glucose uptake in vitro [105]. Therefore, due to low glycemic levels of the mung bean, its use in developing these new products can help to prevent the risk of diabetes.

**Figure 2:**
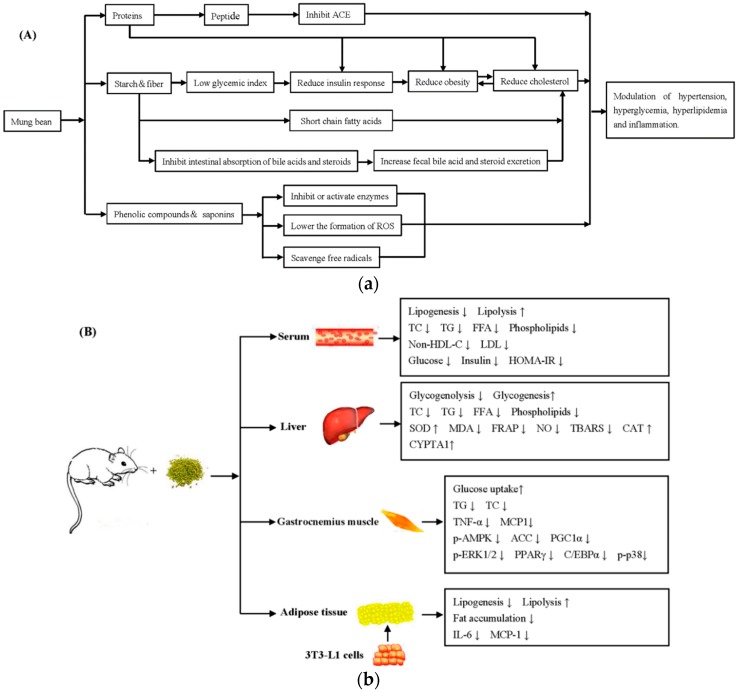
(**A**) Modulation of hypertension, hyperglycemia, and hyperlipidemia and inflammation by the mung bean (including seeds, sprouts, and seed coats) or its active components. (**B**) Schematic diagram depicting the beneficial effects of the mung bean (including seeds, sprouts, and seed coats) or its active components on metabolic responses. The mung bean or its active compounds prevented an increase in serum glucose and lipid concentrations by inhibiting the activities of the related enzymes in a carbohydrate and lipid metabolism, increasing lipolysis in the liver and adipose tissue and decreasing oxidative stress in white adipose tissue, also increasing capacity of energy metabolism in the gastrocnemius muscle. In addition, the expression levels of inflammation-related genes in the liver, muscle, and adipose tissue were inhibited. The mung bean protein was hydrolyzed into peptides with small molecular weight to reduce the blood pressure. ↑ and ↓ sign, after the mung bean or the supplementation of its active compounds, represented an increase and decrease, respectively. (Original).

The results obtained from in vitro and animal studies indicated that the mung bean and its extracts possessed the ability to modulate glucose metabolism effectively (Table 3 and Table 4). Aqueous and ethanolic extracts of the mung bean showed a consequential inhibitory effect on the starch-hydrolyzing enzymes, such as gastrointestinal α-amylase (pancreatic) and α-glycosidase (intestinal) [35,45,64]. This may contribute toward reducing the intestinal absorption of carbohydrates, enhancing insulin sensitivity, and thus reducing body hyperglycemia. From the current research results, it is concluded that vitexin and isovitexin in the mung bean may be the main active components to play a remarkable role in regulating a glucose metabolism. Furthermore, the vitexin and isovitexin contained in the mung bean showed significant inhibitory activities against the formation of advanced glycation end-products induced by glucose or methylglyoxal, with efficacies of over 85% at 100 μM [44]. The animal studies have demonstrated that the whole mung bean and its extracts, including aqueous and ethanolic extracts, can exert beneficial effects on the serum glucose and tissue glycogen content present in diabetic KK-Ay mice and alloxan-induced, streptozotocin-induced, and high fat diet-induced diabetic animal models by enhancing the peripheral utilization of glucose, regulating the activities of glycolytic enzymes, and limiting gluconeogenic formation [21,82,84]. Moreover, the protective effects of the mung bean on the development of diabetes have been investigated in the diabetic db/db mice model, and results showed that the ethanolic extract of the mung bean seed coat significantly improved the insulin sensitivity using the homeostatic model assessment of insulin resistance [66]. Oxidative stress was considered the pathogenesis of diabetes mellitus and its complications, because it plays a key role in insulin resistance and β-cell dysfunction. Recent studies indicated that the aqueous extract of mung bean (0.016 ± 0.001 g γ-amino butyric acid/100 g dried nonfermented mung bean) and the fermented mung bean (a good source of γ-amino butyric acid, 0.122 ± 0.009 g/100 g dried fermented mung bean) exerted a therapeutic protective effect on diabetes by regulating oxidative stress markers and decreasing activities of antioxidant enzymes [53,83]. The potential hypoglycemic mechanism of the mung bean and its active compounds is depicted in Figure 2A,B.

It is noteworthy that the hypoglycemic nature of the mung bean can be significantly affected by the type of processing method. Liyanage et al. evaluated the different effects of the consumption of raw, boiled, and sprouted mung beans on the glucose metabolism of rats in a cholesterol-enriched diet [21]. Results of this study showed that mung beans processed by boiling and sprouting showed higher hypoglycemic and hypolipidemic potential. Similarly, the fermented mung bean had a greater effect on the regulation of blood glucose than the non-fermented mung bean in alloxan-induced hyperglycemic mice [53,83]. Therefore, for the mung bean, the selection of suitable processing methods may be effectively conducive to its health benefits.

### 5.2. Hypolipidemic Properties

Nowadays, hyperlipidemia and atherosclerosis are the main leading causes of cardiac illnesses and deaths. A major risk factor for the development of hyperlipidemia and coronary heart disease is the elevated level of plasma cholesterol. It is crucial to maintain the normal body functions by reducing the elevated serum cholesterol to an adequate level. Studies have shown that consumption of the mung bean is associated with the modulation of a lipid metabolism. It was found that mung bean protein dose-dependently reduced plasma lipids levels, such as (TC), triglyceride (TG), and non-high-density lipoprotein cholesterol total cholesterol (non-HDL-C), in hamsters, which were fed a high-cholesterol diet and partially mediated by up-regulation of 3-hydroxy-3-methyl glutaryl coenzyme A reductase and cholesterol-7α-hydroxylase (CYP7A1), in both transcriptional and translational levels [86,89]. The mechanism underlying the cholesterol-lowering activity of mung bean protein was speculated to increase the fecal bile acids and sterol excretion, as well as decrease the cholesterol absorption and synthesis (Figure 2A,B). In another study, vitexin, isovitexin, and ethanolic extracts of the mung bean significantly lowered the fat accumulation in the in vitro and animal studies by decreasing the expression of lipogenic genes, such as acetyl-CoA carboxylase (a rate limiting enzyme of fatty acid synthesis), the peroxisome proliferator-activated receptor (promoting adipocyte differentiation and fatty acid storage), and the CCAAT/enhancer binding protein alpha (a liver-enriched transcriptional regulator for fat metabolism) [67]. The inhibition of adipogenic gene expression was speculated to be mediated by the activation of the MEK/ERK signaling pathway.

### 5.3. Hepatoprotective Properties

**Figure 3:**
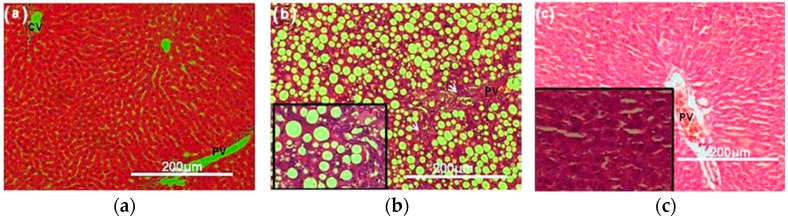
Liver histopathological changes in different experimental animals. (**a**) Normal group, (**b**) rats supplemented with a methionine-choline deficient diet, (**c**) rats receiving the germinated mung bean (1000 mg/kg body weight, for 4 weeks) and supplemented with a methionine-choline deficient diet. Scale bar, 200 μm; White arrow: Fibrosis. Central vein (CV); portal vein (PV) [90].

The liver is a pivotal inflammatory organ that is involved in metabolism, storage, and excretion of metabolites. Liver injury can be caused by different agents, such as alcohol, viruses, and auto-immune diseases [106,107]. The whole mung bean and the germinated mung bean have proved to be an effective hepatoprotective agent, which is able to decrease the liver enzyme activities and liver histopathology in a dose-dependent manner [26]. Figure 3 shows the liver histopathological changes in experimental animals with a germinated mung bean supplemented diet (1000 mg/kg body weight, for 4 weeks), in comparison to that of the rats supplemented with a methionine-choline deficient diet (non-alcoholic fatty liver disease) and the normal group. Diets containing the germinated mung bean exhibited a good protective effect against liver injury caused by the hepatic deposition of excess lipids, demonstrating the vascularization of hepatic tissue and absence of steatosis and inflammatory infiltrates. In addition, the involvement of the reactive oxygen species (ROS), oxidative stress, and end/by products of oxidative stress are important mediators that worsen the non-alcoholic fatty liver disease [108]. Recently, it was reported that the germinated mung bean could ameliorate oxidative stress to prevent non-alcoholic steatohepatitis from steatosis by enhancing antioxidant gene expression and reducing mitochondrial ROS generation [90]. Besides this, the aqueous extract of the mung bean showed a stronger hepatoprotective effect on the acetaminophen-induced acute liver injury model in rats than other pulses, including adzuki bean, black bean, and rice bean [93].

Previous studies have demonstrated that several components occurring in legumes could be responsible for liver-protective effects, such as bioactive compounds and dietary fiber, as well as constituent proteins, due to their composition of specific amino acids and bioactive peptides [109,110]. Watanabe et al. evaluated the effect of the mung bean protein isolate on hepatic TG accumulation in mice fed with a high-fat diet to elucidate its potential ability to prevent non-alcoholic fatty liver disease onset and progression [91]. In this study, it was found that the mung bean protein isolate could alleviate hepatic TG accumulation and suppress hepatic inflammation and fibrosis via a mechanism that is independent of weight loss in a non-alcoholic steatohepatitis model. The peptides from the enzymatically digested mung bean protein isolate suppressed the expression of de novo lipogenesis-related genes (*Srebf1*, *Fasn*, and *Scd1*) in primary hepatocyte cultures, suggesting that the mung bean protein isolate acted directly on the liver to decrease TG accumulation. Besides the non-alcoholic fatty liver disease, alcohol-induced oxidative stress is another severe liver injury. Aqueous extracts of the germinated and fermented mung bean significantly decreased the serum alanine aminotransferase (ALT), Aspartate aminotransferase (AST), TC, TG, nitric oxide (NO), and malondialdehyde (MDA)and increased the ferric ion reducing antioxidant power (FRAP) and uperoxide dismutase (SOD) activities in the ethanol-induced liver injury [52]. Similarly, in another study, the flavonoid fraction (vitexin and isovitexin) also exhibited an excellent hepatoprotective activity by suppressing the accumulation of hepatic lipids and maintaining the antioxidant status in the acute alcohol-induced liver injury model [92]. Interestingly, a better hepatoprotective effect was observed in the germinated and fermented mung beans or their extracts as compared to the mung bean [52,90].

### 5.4. Antihypertensive Properties

The ACE inhibitor is one of the main drugs that plays an important role in regulating blood pressure. ACE inhibitory peptides derived from food proteins could be safer and milder without side effects than synthetic ACE inhibitor drugs [111]. As described in Section 4, the peptides obtained from the alcalase hydrolysate of mung bean protein isolates exhibited high ACE inhibitory activity in vitro [25,62]. For the purpose of exerting an antihypertensive effect in vivo, the ACE inhibitory peptides must be absorbed in their intact form from the intestine and further resist the degradation by plasma peptidases to reach their target sites after oral administration. Thus, the in vitro ACE inhibitory activity of these peptides is not always consistent with their antihypertensive activity in vivo. The mean systolic blood pressure of 2, 4, 6, and 8 h in spontaneously hypertensive rats (187.9 ± 8.7 mmHg) significantly decreased after single oral administration of the mung bean protein hydrolysate (prepared with alcalase at 2 h hydrolysis time) at a dose of 600 mg/kg of body weight [94]. The systolic blood pressure was decreased by 30.8 mmHg at 6 h after administration, and the antihypertensive effect lasted for at least 8 h.

As it is known, a large number of peptides and other metabolites were produced during germination [7]. Sprouts, or their bioactive components, such as peptides, may have beneficial properties and may be useful for hypertension management. Highdose aqueous extracts of raw, dried, and enzyme-digested mung bean sprouts (600 mg peptide/kg body weight) significantly reduced the 6–9, 3–6, and 3–9 h of systolic blood pressure in spontaneously hypertensive rats after a single intragastric administration [95]. A long-term intervention test was also carried out in the same study. The results showed that, after intragastric administration of 30 mL/day of reconstituted raw and dried aqueous extracts (600 mg peptide/mL) for 4 weeks, the systolic blood pressure of the rats was significantly reduced from week 1–4 and week 2–4, respectively. The freeze-dried powder of sprouts (or approximately 488 mg peptide/day) showed no significant effects on the systolic blood pressure of the treated rats after 4 weeks of intragastric administration. This might be due to the higher concentration of peptides in water extraction, which was more contributed to the reduction of blood pressure. This result was also strongly influenced by the intragastric dosage. Accurate research is needed to confirm these speculations. These results indicated that the protein hydrolysates of the mung bean and its sprouts might be utilized for physiologically functional foods in the prevention and management of hypertension.

### 5.5. Anticancer Properties

Research showed that the choice of food consumed during life may alter the probability of carcinogenesis in every stage of the cancer process in a way that can reduce the risk, but usually in a favorable direction, although this disease is related to different factors, such as genetic susceptibility, smoking, obesity, chronic inflammation, immunosuppression, and radiation [112]. There is significant evidence suggesting the links between bean-rich diets and reduced risk of numerous types of cancer [113,114]. Evidences from the in vitro studies suggested that peptides, proteins, and phenolic acids found in the mung bean exerted the dose-dependent anti-proliferative effects against various cancer cell lines, such as human breast adenocarcinoma cells (MCF-7 and MDA-MB-231), digestive system cancer cells (CAL27, AGS, HepG2, SW480, and Caco-2), and leukemia cells (HL-60) (Table 3).

To date, the ingredients in the mung bean clearly have an anti-proliferative effect on cancer cells, but the specific regulatory mechanism in the prevention of cancer cells has not been fully understood. The cell-mediated immune response is an important aspect of host resistance to infection and cancer. The current study showed that the methanolic extract of the mung bean is found to act as a potent inducer for apoptosis in treated human cancer cells via a caspase-dependent apoptosis pathway (both an extrinsic and intrinsic pathway) and maybe by a caspase independent pathway [72]. Furthermore, the acidified methanol extracts of the mung bean sprout can induce apoptosis and cell cycle arrest via cell-type specific interactions [72]. In this study, it was concluded that the Cdk-inhibitor proteins (p21, p27, and p53), tumor necrosis factor alpha (TNF-α) and interferon-β (IFN-β) may be key regulatory factors associated with the apoptotic and cell cycle slowing/arresting. In addition, the in vivo study suggested that aqueous extracts of the fermented mung bean can delay the formation of breast cancer and reduce the mitotic division of the tumor through the stimulation of T cell cytokine production (IL-2 and IFN-γ) and cytotoxicity [96].

### 5.6. Immunomodulatory Activity

The immune response is a complex series of steps, aiming at identifying, attacking, and eliminating organisms and substances that invade our system or cause disease [115]. Traditionally, immune processes have been divided into two broad, but interconnected, subsystems on the basis of their functions in host defense, namely innate and adaptive immunity [116]. The innate arm of immunity is composed of those immunological effectors that provide robust, immediate, and nonspecific immune responses [117]. The macrophage is one of the major immunocytes, and its activation occupies a unique niche in mediating innate immune responses via the increase of signaling chemical (NO) production and secreting cytokines, such as TNF-α, interleukin-6 (IL-6), and interleukin 1 beta (IL-1β). [118]. In contrast, the adaptive immune system is organized around two classes of specialized lymphocytes, T and B cells, displaying an extremely diverse repertoire of antigen-specific recognition receptors. At present, the effect of the mung bean and its bioactive compounds on immune effects is mainly reflected in the innate immune response [119].

The mung bean is rich in fiber (Table 1), mainly composed of several non-starch polysaccharides. The beneficial role of non-starch polysaccharides in immunomodulation activity is increasingly being recognized [120]. Polysaccharides isolated from the mung bean using water and alkali have been shown to activate macrophages [57,58]. Dai et al. found that treatment with 200 μg/mL verbascose from the mung bean exhibited the most significant immunomodulatory activity in strengthening the ability of peritoneal macrophages to devour neutral red and promote the production of NO and immune reactive molecules (IL-1β, IL-6, interferon-α(IFN-α), and interferon-γ (IFN-γ) [73]. Furthermore, after administration of verbascose at a medium dose of 90 mg/kg body weight for 8 consecutive days, the index of the spleen, activity of lysozyme in spleen and serum, hemolysin level in serum, and swelling rate of earlap in the delayed type of hypersensitivity of immunosuppressed mice were significantly increased.

Inflammation has been recognized as a risk factor for a number of health problems. There is strong evidence suggesting that chronic inflammation is a major factor contributing to the development and progression of autoimmune disease, atherosclerosis, and some cancers [121]. Inflammation is a host’s defense mechanism with the sequential release of NO and pro-inflammatory cytokines by activating the cellular immune responses. Thus, a variety of inflammatory diseases may be prevented or suppressed by inhibiting the overproduction of inflammatory mediators, especially pro-inflammatory cytokines. In China, mung bean seeds are not only used in all kinds of cuisines but are also used in traditional medicine to treat heat stroke associated with thirst, fever, and irritability, stated by the ancient Chinese medical text “Compendium of Materia Medica”. Moreover, the beneficial health effects of the mung bean are believed to be closely associated with the inflammatory response. Current research has also shown that the inflammatory response can be modulated by means of specific bioactive components of the mung bean [122]. Suk-Jun et al. reported that the pro-inflammatory cytokines, including IL-1β, IL-6, interleukin 12β (IL-12β), TNF-α, and inducible nitric oxide synthase (iNOS) were significantly decreased in the lipopolysaccharide (LPS)-stimulated macrophages (J774) when treated with the ethanolic extract of the mung bean [77]. Zhang et al. evaluated the anti-inflammatory properties of acetone-water mung bean extracts, vitexin, and isovitexin and the mixture of vitexin and isovitexin on the expression of the IL-**1**β, IL-6, and cyclooxygenase-2 (COX-2) mRNA in the LPS-stimulated RAW 264.7 mouse macrophage cells [34]. The results indicated that, although vitexin and isovitexin might play an important role in suppressing the mRNA expression of pro-inflammatory cytokines, other components, such as phenolic acids, also contributed to the overall anti-inflammatory effect of the mung bean. In addition, there was no synergetic effect between vitexin and isovitexin for their anti-inflammatory activities. Antigen-specific T helper cells are key immune regulators that secrete various cytokines affecting adaptive immunity. However, excessive Th cell activity occurs occasionally during severe inflammation, which leads to the development and progression of autoimmune diseases, atherosclerosis, and some cancers [123]. Mung bean saponin was found to be effective in preventing the antigen-specific activation of Th cells by inhibiting cell proliferation and cytokine secretion [75]. The possible mechanism was that the saponins from the mung bean directly inhibited Th cell proliferation by blocking the G_1_ to S phase cell-cycle transition. The decreased expressions of cyclin D1 and cyclin E, and constitutive expression of p27^KIP1^, may be the reason for the blocking of the cell cycle by the saponins.

It has been reported that the mung bean has a positive anti-inflammatory effect against sepsis, arthritis, and arachidonic acid-induced ear edema, and significantly inhibits the expression of pro-inflammatory factors, such as TNF-α and IL-1β in animal studies [22,76,97]. In addition, Inhae et al. suggested that the functional compounds in the mung bean ethanol extracts, such as vitexin and isovitexin, may decrease pro-inflammatory cytokine-induced lipogenesis via anti-inflammatory mechanisms and the MEK/ERK pathway in the KK-Ay mouse model [67]. The results showed consistency between in vitro and in vivo anti-inflammatory studies. In fact, the concept of type 2 diabetes as an inflammatory disease has recently emerged and appears to be confirmed by growing evidence [124]. Numerous studies have shown that obesity is a proinflammatory state associated with systemic low-grade inflammation, which leads to insulin resistance and metabolic disorders [125]. Thus, the anti-inflammatory effects of active ingredients in the mung bean may play an important role in the treatment of many diseases, such as diabetes and obesity. In general, the mung bean possesses various bioactive compounds, including polyphenols and saponins, inhibiting the pro-inflammatory gene expression.

### 5.7. Anti-Melanogenesis Properties

Tyrosinase is a key rate-limiting enzyme in melanogenesis. Its abnormal expression can lead to various dermatological diseases. In a previous study, among 16 legumes, the ethanolic extract of the mung bean showed the highest tyrosinase inhibition ability [81]. When using L-3,4-dihydroxyphenylalanine as the substrate for mushroom tyrosinase, the ethyl acetate extract fractions of the mung bean showed the highest inhibitory activity compared with the other three fractions, i.e., dichloromethane extract, n-butanol extract, and residual extract fractions [78]. In this study, two pure compounds separated from the ethyl acetate extract fraction were identified as vitexin and isovitexin. In another study, an ethyl acetate extract of mung bean seeds and its sprouts was also investigated for inhibitory effects against melanogenesis using B16F1 melanoma cells [79]. The results showed that inhibitory effects against melanogenesis were especially apparent for 10 μg/mL ethyl acetate fractions of mung bean sprouts (germinated for 1 day), with values of 56.5% in B16F1 melanoma cells treated with a 200 nM α-melanocyte-stimulating hormone. Identification of main components was also confirmed as vitexin and isovitexin. Vitexin was proved to exhibit a higher inhibitory effect on melanogenesis than isovitexin. In addition, proanthocyanidins and condensed tannins from the mung bean seed coat were also found to have a good inhibitory effect on cellular tyrosinase activity and melanogenesis of B_16_ mouse melanoma cells [24,80]. The interactions of proanthocyanidins or condensed tannins from the mung bean seed coat with tyrosinase, driven by hydrogen bonding and hydrophobic force, according to molecular docking, were considered as a significant mechanism for explaining the inhibition.

### 5.8. Other Health Benefits

In addition to the above, there are some other health benefits that deserve attention. In China and other Asian countries, it is well-known that the mung bean is a functional food with the ability to detoxify. However, the mechanism underlying its detoxifying effect remains unclear. Gao et al. evaluated the effects of single (1 g/kg body weight) and multiple (1 g/kg body weight for 7 days) oral administration of the ethanolic extract of the mung bean on the pharmacokinetics of aconitine (highly toxic diester-diterpene) in rats [126]. The results showed that an ethanolic extract of mung bean significantly altered the pharmacokinetic parameters of aconitine and its detoxifying mechanism to prevent the intestine absorption of aconitine, not to alter the metabolism of aconitine. Similarly, the various biotoxicity effects of aluminum (Al) has been associated with the development of some cardiovascular diseases. It was found that the mung bean polyphenol extract (200 mg/kg body weight for 12 weeks) had a cardiac protective effect in Al-induced cardiotoxicity rats, which might take place through a ROS-triggered c-Jun N-terminal kinase (JNK) and NF-κB-mediated caspase pathways [127]. Besides being a major secondary metabolite component in the mung bean, vitexin and isovitexin (6 mg/kg body weight for 7 days) also showed a significant protective effect against isoproterenol-induced myocardial ischemia in rats [128].

### 5.9. Health Benefits of the Mung Bean on Clinical Trials

Despite the health benefits of the mung bean described above in in vitro and animal experiments, only a few clinical trials performed on the mung bean are available in the existing literature (Science Citation Index). Lack of clinical trials is due to many factors, such as ethical issues, limited commercial support, and the complex chemical composition of the mung bean. Currently, the available clinical trials have mainly focused on the improvement of plasma, glucose, and lipids.

A randomized clinical trial carried out to assess the insulin and glycemic responses of 18 healthy humans of the mung bean noodle (made of pure starch) [129]. The results showed that cooked mung bean noodles exhibited low metabolic responses, similar to those of raw starches from wheat, manihot, and smooth peas. High amylose content (32%) in mung bean starch appeared to be responsible for the low glucose and insulin plasma responses encountered. The similar results were found in clinical trials of eight healthy nondiabetic men [130]. The mean (± SEM) glycemic index of mung bean starch was lower (51 ± 13) than that of cornstarch (95 ± 18). The net posthepatic appearance of glucose from mung bean starch was significantly lower (35.6 ± 4.6% of the load, *p* < 0.001) than that from glucose and cornstarch, even 4.5 h postprandially, which was assessed by a euglycemic hyperinsulinemic clamp plus oral carbohydrate loading. These studies reported making mung bean an attractive option for diabetic patients.

Furthermore, the mung bean protein isolate is mainly composed of 8 S globulins, with a structure very similar to that of soybean β-conglycinin, which has been reported to have physiologically beneficial effects on lipid and glucose metabolism [131,132]. A recent double-blind, placebo-controlled clinical trial of 44 healthy subjects showed that, after consumption of mung bean protein isolates 3.0 g/d for 8 weeks, the insulin levels and homeostatic model assessment of insulin resistance values significantly decreased and the plasma glucose levels showed a downtrend, though not significantly [133]. Lacking a beneficial effect of the mung bean protein isolate on blood glucose concentrations may be attributed to the exclusion of volunteers with abnormal blood glucose concentrations in this study. Interestingly, another double-blind placebo-controlled clinical trial was carried out to confirm the positive effects of mung bean protein on a glucose metabolism in 45 prediabetes patients (fasting plasma glucose level of 110–125 mg/dL or 2 h plasma glucose level (2 h PG) using the 75 g glucose tolerance test of 140–200 mg/dL) [134]. In this study, the subjects in the test group (*n* = 23) were instructed to consume a total of 2.5 g mung bean protein twice daily for 12 weeks. Here, the mung bean protein was shown to stabilize fasting plasma glucose and insulin levels, as compared to the placebo group. Triglyceride levels significantly decreased in the subjects with hyperlipidemia. These findings indicated that mung bean protein might improve insulin sensitivity by decreasing the accumulation of visceral fat. These effects were particularly observed in obese subjects. In addition, a mung bean-based oral rehydration solution has been successfully used for the treatment of children aged 3 months to 5 years with moderate dehydration and acute diarrhea [135]. There was no a statistical difference between the 60 g/L of mung bean-based rehydration solution and the 20 g/L standard glucose electrolyte solution in the percentage of patients recovering from diarrhea within the 72 h study period.

## 6. Conclusions, Limitations of Current Knowledge, and Future Perspectives

The mung bean contains abundant nutrients and bioactive compounds, especially polyphenols, polysaccharides, and polypeptides, and it possesses various pharmacological properties. The in vitro and in vivo studies presented in this review have been provided to support the theory that the mung bean has been associated with health benefits, such as hypoglycemic and hypolipidemic effects and antihypertensive, anticancer, anti-melanogenesis, hepatoprotective, and and immunomodulatory activities. Given their health and nutritional benefits, the consumption of mung bean-based functional products, as well as nutraceuticals, could be considered an alternative food, not just in Asian countries but also in other countries.

However, there are still important gaps in our knowledge regarding the bioactive compounds and biological activities of the mung bean. To date, numerous studies mainly focus on the aqueous and various organic solvent extracts of the mung bean. Although vitexin and isovitexin are identified as the main functional components in many published studies, further research is also needed to unravel other main functional components relevant to the health benefits and highlight the synergistic multi-component effects of the mung bean on biological functions. In addition, the mung bean is usually subjected to processing before consumption, involving mechanical force, thermal treatment, and a biotechnical process. As a result, the functional qualities of the mung bean will be changed due to the different types of processing. However, grain functionality is directly linked to the type of processing applied to it. Currently, there is a limited understanding of how food processing methods affect the physiochemical properties of the mung bean and the effects of various mung bean fractions on relevant health outcomes.

Though the in vitro and in vivo studies from recent years have built a consensus that the mung bean and its major constituents exert beneficial health effects, the mechanisms involved in disease prevention is imperative to unravel. For example, whether the regulation of mediators and transcription factors, such as peroxisome proliferator-activated receptors (PPARs), the adenosine monophosphate-activated protein kinase (AMPK), and the NF-κB signaling pathway, can contribute to the therapeutic effects of the mung bean or not. In addition, growing evidence has revealed reciprocal interactions between the gut microbiota and functional food components and its consequences on human health [136,137]. Thus, the mung bean-involved mechanisms of gut microbiota may offer possible new routes for therapeutic interventions.

## Figures and Tables

**Table 1:** Polyphenol composition and relative content in the mung bean (fresh weight).

Class	Subclass	Compound	Content	References
Flavonoids	Anthocyanins	Cyanidin-3-glucoside	256.32–476.53 μg/g	[35]
		Peonidin-3-glucoside	5.43- 8.42 μg/g	[35]
		Pelargonidin-3,6-malonylglucoside	8.53–12.49 μg/g	[35]
		Pelargonidin-3-glucoside	147.62–350.71 μg/g	[35]
	Flavonols	Quercetin	0.17–16.2 mg/100 g	[36,37,38]
		Myricetin	0.03–4.26 mg/100 g	[36,37]
		Kaempferol	0.07–6.13 mg/100 g	[36,37]
	Flavanols	Catechin	4.39–35.36 mg/100 g	[36,37]
	Flavones	Vitexin	17.04–62.37 mg/100 g	[36,37]
		Isovitexin	22.63–73.64 mg/100 g	[36,37]
		Isovitexin-6″-O-α-L-glucoside	1.70 mg/g	[39]
		Luteolin	0.36 mg/100 g	[38]
	Isoflavonoids	Dulcinoside	0.13 mg/g	[39]
Phenolic acids	Hydroxycinnamic acid	p-Coumaric acid	8.17–38.34 mg/100 g	[36,37]
		Caffeic acid	1.37–38.72 mg/100 g	[36,37]
		t-ferulic acid	8.02–54.77 mg/100 g	[36,37]
		Chlorogenic acid	0–26.55 mg/100 g	[36]
		Sinapic acid	22.46 mg/100 g	[38]
	Hydroxybenzoic acid	Gallic acid	1.32–9.47 mg/100 g	[36,38]
		Syringic	0–220.29 μg/g	[35]
		Gentisic	25.39–138.45 μg/g	[35]

**Table 2:** Polysaccharides isolated from the mung bean and their bioactivities.

Compound Name	Molecular Weight (kDa)	Monosaccharide Composition (Molar Ratio, %)	Bioactivities	Reference
MP1	83	Fuc:Ara:Xyl:Man:Gal:Glu = 8.3:2.2:67.2:20.1:2.3	Antioxidant activities	[55]
MP2	45	Rha:Fuc:fucose:Ara:Xyl:Man:Gal:Glu = 31.8:3.5:16.7:4.6:11.7:29.1:2.5	Antioxidant activities	[55]
MEMP-1	Nd ^1^	Ara:Man:Gal = 0.12:1.0:0.5	Radicals scavenging activity	[56]
MEMP-2	Nd	Rha:Ara:Man:Gal = 1:0.3:0.3:0.6	Radicals scavenging activity	[56]
MWP-1′	68.4	Rha:Ara:Man:Galin = 0.4:2.6:5.3:0.7	Immunoregulatory activities	[57]
MWP-2′	52.4	Ara:Man:Gal:Glc = 0.5:1.4:2.1:0.4	Immunoregulatory activities	[57]
MAP-1	94.2	Rha:Ara:Glu:Gal:GalA = 1.1:0.4:0.7:0.5:0.3	Antioxidant and immunoregulatory activity	[58]
MAP-2	60.4	Xyl:Rha:Gal:Glu:GalA = 0.4:1.4:1.6:0.5:0.2	Antioxidant and immunoregulatory activity	[58]
WSP	20–300	Ara:Man:Gal:Glu = 3.5:12:66:18.5	Nd	[28]
HWSP	15–150	Rha:Ara:Xyl:Man:Gal:Glu = 2.5:34:5:8:33.5:17	Immunomodulatory activity	[28]
Pectins	40–1200	Rha:Ara:Xyl:Gal:Glu = 2.6:46:8:26.4:17	Immunomodulatory activity	[28]
Hemicellulose A	15–350	Ara:Xyl:Man:Gal:Glu = 8.5:23:2:9:57.5	Nd	[28]
Hemicellulose B	100–1800	Rha:Ara:Xyl:Gal:Glu = 2.5:43:7:28:19.5	Immunomodulatory activity	[28]
Arabinogalactan	1200	Rha:Ara:Xyl:Gal:Glu = 5:60:2:32:1	Macrophage activation	[59]

^1^ Nd: No data were found.Mung bean polysaccharide 1 (MP1); mung bean polysaccharide 2 (MP2); microwave extraction mung polysaccharide-1 (MEMP-1); microwave extraction mung bean polysaccharide-2 (MEMP-2); water-soluble mung bean polysaccharide-1′ (MWP-1′); water-soluble mung bean polysaccharide-2′ (MWP-2′); alkali-extractable mung bean polysaccharide-1 (MAP-1); alkali-extractable mung bean polysaccharide-2 (MAP-2,); water-soluble polysaccharide (WSP); hot water soluble polysaccharide (HWSP); rhamnose (Rha); fucose (Fuc); arabinose (Ara); xylose (Xyl); mannose (Man); galactose (Gal); glucose (Glu).

**Table 3:** In vitro studies of the mung bean and its active compounds in health benefits.

Health Benefits	Model	Type of Extract/Constituents	Dose/Reaction System	Experimental Outcome	Reference
Hypoglycemic properties	Biochemical tests	Lignans and flavonoids	20 μL/1650 μL	Inhibited the activity of α-glucosidase	[64]
	Biochemical tests	Vitexin and isovitexin	500 ppm, 100 μm	Inhibited the formation of advanced glycation end products	[44]
	Biochemical tests	Phenolic compounds	50 μL/200 μL, 1 mL/3 mL	Inhibited the activity of α-glucosidase and the formation of advanced glycation end products	[35]
	Biochemical tests	Aqueous extracts of raw, boiled, and sprouted mung bean	20 μL/220 μL, 40 μL/260 μL	Inhibited the activity of α-glucosidase and α-amylase	[21]
	Biochemical tests	Aqueous extract of bioprocessed mung bean	800 μL/6 mL	Inhibited the activity of α-Amylase	[65]
	Biochemical tests	Ethanolic extracts of whole mung bean, cotyledon, and hull	0.1 mL/1 mL	Inhibited the activity of aldose reductase	[45]
	Biochemical tests	Ethanolic extract of mung bean seed coat	5 mg/mL	Inhibited the activity of α-glucosidase	[66]
Hypolipidemic properties	3T3-L1 preadipocytes	Vitexin and isovitexin	25, 50 and 100 μM	Decreased fat accumulation Lowered inflammatory cytokines, IL-6 and MCP-1	[67]
Antihypertensive properties	Biochemical tests	Mung bean protein hydrolysates	5, 7.5, 10, 12.5, 15, 20 and 25 μg/mL	Exhibited ACE-I inhibitory activity	[25]
	Biochemical tests	Vicilin protein (storage protein of mung bean) hydrolysate	0.2–1.0 mg/mL	Exhibited ACE-I inhibitory activity	[23]
	Biochemical tests	Mung bean protein hydrolysate	100 μg/mL	Exhibited ACE-I inhibitory activity	[63]
	Biochemical tests	Mung bean protein hydrolysate	10 mg protein/mL	Exhibited ACE-I inhibitory activity	[62]
Anticancer properties	Human breast adenocarcinoma cells (MCF-7), human cervical cancer cells (Hela)	Proteins isolated from mung bean aqueous extract	62.5, 125, 250, 500 and 1000 μg/mL	Exhibited the anti-proliferation activities	[68]
	Human breast adenocarcinoma cells (MCF-7 and MDA-MB-231)	vicilin protein (storage protein of mung bean) hydrolysate	10, 25, 50, 75 and 100 mg/mL	Exhibited the anti-proliferation activities	[23]
	Human hepatoma cells (Bel-7402)	Mungoin- a novel mung bean protease inhibitor	10, 50, 100 and 200 μM	Exhibited the anti-proliferation activities	[69]
	Digestive system cancer cells (CAL27, AGS, HepG2, SW480 and Caco-2), prostate cancer cells (DU145), ovary cancer cells (SK-OV-3), breast cancer cells (MCF-7), and leukemia cells (HL-60)	Phenolics	0.125, 0.25, 0.5, 1, 2 and 5 mg/mL	Exhibited the anti-proliferation activities	[70]
	Human pulmonary carcinoma cell, human gastric carcinoma cells (SNU-601)	Aqueous, ethyl acetate, methanol, n-hexane, n-butanol extracts of mung bean seeds and sprouts	Nd ^1^	Exhibited the anti-proliferation activities	[71]
	Cervix adenocarcinoma cells (HeLa; ATCC CCL-2), hepatocellular carcinoma (HepG2; ATCC HB-8065)	Methanol Extracts of mung bean sprouts	9.37 to 300 mg/mL, 10.25 to 164 mg/mL, 3.12 to 100 mg/mL, 0.31 to 10 mg/mL	Increased levels of anticancer cytokine (TNF-α and IFN-β) Induced IFN-γ and inhibited IL-4 production Induced apoptosis in HeLa and HepG2 cells Induced cell cycle arrest in HeLa Induced cdk-inhibitor proteins (p21, p53, and p27) in HeLa cells Induced only p53 in HepG2 cells	[72]
Immunomodulatory activity	Murine macrophage RAW264.7 cells	Verbascose	25, 50, 100, 200, and 400 μg/mL	Enhanced the ability of devouring neutral red of peritoneal macrophages Promoted the release of NO and immune reactive molecules, IL-6, IL-1β, IFN-α, and IFN-γ	[73]
	Murine macrophage RAW264.7 cells	Arabinogalactan	10, 50, 100, and 200 μg/mL	Induced the release of NO, TNF-α, IL-6, and IL-1β Increased phagocytic capability of macrophages	[59]
	Male Balb/c mice splenocyte (8–10 week-old)	Aqueous extract of fermented mung bean	2.3 mg/mL	Enhanced splenocyte proliferation Increased serum IL-2 and ŽIFN-γ concentrations	[74]
	Murine macrophage RAW264.7 cells	Water-extractable polysaccharides from mung beans	50, 100 and 200 μg/mL	Stimulate the production of NO, TNF-α and IL-6	[57]
	Murine macrophage RAW264.7 cells	Alkali-extractable polysaccharides from mung beans	50, 100 and 200 μg/mL	Stimulated the production of NO, TNF-α and IL-6	[58]
	Male Wistar splenocytes (8 week-old), murine macrophage RAW264.7 cells	Water soluble (cold and hot water, 55 °C), EDTA soluble (0.5%, Pectins), alkali-soluble (10%, Hemicellulose A and B) polysaccharides isolated from mung beans	0.1–100 μg/mL, 50–1000 μg/mL	Enhanced splenocyte proliferation Increased the production of NO	[28]
	T helper cells (transgenic OT-II mice)	Saponins	50 and 100 μg/mL	Inhibited Th cell proliferation	[75]
	Murine macrophage RAW264.7 cells	Vitexin and isovitexin	100 μg/mL	Inhibited the expression of IL-1β, IL-6, and COX-2 mRNA	[34]
	Murine macrophage RAW264.7 cells	Aqueous extracts of untreated, germinated, and fermented mung beans	2.5 and 5 mg/mL	Decreased level of NO	[22]
	Murine macrophage RAW264.7 cells and human monocyte U-937 cells	Aqueous extract of mung bean seed coat	0.1, 0.2, 0.8, 4, 8, and 15 mg/L	Reduced both intra- and extracellular HMGB1 levels in endotoxin-stimulated macrophages Stimulated HMGB1 protein aggregation Facilitated the formation of microtubule-associated protein-1-light-chain-3-(LC3-) and the production of LC3-II	[76]
	Biochemical tests	Ethanolic extracts of whole mung bean, cotyledon, and hull	1 mL/5 mL	Inhibited the activity of protease	[45]
	Macrophages cells (J774)	Ethanolic extract of mung bean	3.7 mg/mL	Decreased the mRNA expression of IL-1β, IL-6, IL-12β, TNF-α, and iNOS	[77]
Anti-melanogenesis properties	Biochemical tests	Vitexin and isovitexin	10 and 15 μM	Inhibited tyrosinase activity	[78]
	Mouse melanoma cells (B16F1)	Vitexin and isovitexin	10–250 μg/mL	Inhibited melanogenesis	[79]
	Mouse melanoma cells (B16)	Tannins	50, 100, 200, and 400 μg/mL	Inhibited cell proliferation, cellular tyrosinase activity, and melanogenesis	[24]
	Biochemical tests	Antityrosinase	20, 40, 60, 80, 120, 160, and 200 μg/mL	Inhibited the monophenolase and diphenolase activities	[80]
	Biochemical tests	Ethanolic extract of mung beans	15 mg/mL	Inhibited tyrosinase activity	[81]
	Biochemical tests	Aqueous, ethyl acetate, methanol, n-hexane, n-butanol extracts of mung bean seeds and sprouts	Nd	Inhibited tyrosinase activity	[71]

^1^ Nd: No data were found. Interleukin-6 (IL-6); monocyte chemoattractant protein-1 (MCP-1); interleukin 1 beta (IL-1β); cyclooxygenase-2 (COX-2); nitric oxide (NO); high mobility group box-1 (HMGB1); interleukin 12β (IL-12β), tumor necrosis factor alpha (TNF-α); inducible nitric oxide synthase (iNOS); interferon-α (IFN-α); interferon-γ (IFN-γ); interleukin-4 (IL-4); interferon-β (IFN-β); interleukin-2 (IL-2); angiotensin-converting enzyme (ACE); ethylenediaminetetraacetic acid (EDTA).

**Table 4:** Animal studies on the mung bean and its active compounds in health benefits.

Health Benefits	Model	Dose and Duration	Experimental Outcome	Reference
Hypoglycemic properties	Wistar rats in a cholesterol-enriched diet	Raw, boiled, and sprouted mung beans (30%) in diet supplementation for 5 weeks	↓ Blood glucose, insulin, TG, non-HDL-C, HDL-C ↑ Liver weight, cecal weight, fecal matter weight	[21]
	STZ-induced diabetes rats	Fermented mung bean seed coat (100 and 200 g/kg) in diet supplementation for 21 days	↓ Feed intake, body weight, ↓ serum glucose, TC, TG ↑ Liver weight, cecum weight	[82]
	Alloxan-induced diabetes mice	Aqueous extracts of fermented and nonfermented mung bean (200 and 1000 mg/kg) for 10 days	↓ Blood glucose, TG, LDL, NO level	[83]
	Diabetic db/db mice	Ethanolic extract of mung bean seed coat (1%) in diet supplementation for 7 weeks	↓ Fasting serum glucose, blood glycated hemoglobin, HOMA-IR ↓ Lipid peroxides ↑ SOD activity, CAT activity, GSH-Px activity	[66]
	Diabetic KK-Ay mice	Ethanolic extracts of mung bean sprout and mung bean seed coat (2 and 3 g/kg) for 5 weeks	↓ Body weight, blood glucose level, C-peptide level, glucagon level, TC	[84]
Hypolipidemic properties	Fructose-loaded spontaneously hypertensive rats	Mung bean sprouts (30%) in diet supplementation for 46 days	↓ TG, TC ↓ Heat rate	[85]
	Hamsters in a cholesterol-enriched diet	Mung bean (1% and 2%) in diet supplementation for 6 weeks	↓ Plasma TC, TG, non-HDL-C, non-HDL-C/HDL-C, and TC/HDL-C, liver cholesterol ↑ Coprostanol, total neutral sterol, deoxycholic acid, chenodeoxycholic acid, ursocholic acid, total acidic sterol, cholesterol intake, total sterol excretion ↓ Apparent cholesterol absorption ↑ The protein level of CYP7A1	[86]
	High-fat-diet-induced rats	The juice of mung bean sprout (0.67 and 1.34 mg/200 g) for 28 days	↓ TC, TG, LDL ↑ HDL	[87]
	Balb/c mice in a cholesterol-enriched diet	Aqueous extracts of fermented and nonfermented mung beans (200 and 1000 mg/kg) for 2 weeks	↓TC, TG, LDL, ALT, ALP ↓ MDA, FRAP, NO ↑ HDL, SOD	[53]
	High-fat diet-induced male C57BL/6 mice	Mung bean protein isolate in diet supplementation (26.35%) for 4 weeks	↓ body weight, epididymal, perirenal, and subcutaneous adipose weights, hepatic triglyceride ↑ Glucagon-like peptide-1, secondary/primary bile acid ratio, phylum Bacteroidetes ↓ Abundance of the Firmicutes	[88]
	Hamsters in a cholesterol-enriched diet	Mung bean protein isolate in diet supplementation (1% and 2%) for 6 weeks	↓ TC, TG, non-HDL-C, non-HDL-C/HDL-C, and TC/HDL-C ↑ Sterol excretion ↓ The cholesterol absorption ↑ The production of mRNA3-hydroxy-3-methyl glutaryl coenzyme A reductase, CYP7A1	[89]
Hepatoprotective	Hamsters in a casein hypercholesterolemic diet	Cooked and germinated mung beans (as 22.1% protein source) in diet supplementation for 28 days	↓ Relative liver weight, non-HDL-C, AST, ALT ↑ Fecal cholesterol	[26]
	Methionine and choline-deficient diet induced steatohepatitis rats	Germinated mung bean power (500 and 1000 mg/kg) for 4 weeks	↓ Lipid deposition, inflammatory infiltrate ↑ Vascularisation of the hepatic tissue ↓ serum ALT and AST activities, nitrite/nitrate, TBARS, and GSH levels ↑ GSH, SOD, CAT, GPx, GR, G6PDH levels in the liver ↓ TBARS level in the liver ↑ mRNA levels of MnSOD, Cu/ZnSOD, GPx1, CAT, and G6DPH in liver ↓ Serum TC, TG, FFA, Phospholipids ↓ Liver TC, TG, FFA, Phospholipids ↓ Inflammatory cytokines TNF-α, IL-10, IL-1β, IL-6 levels in serum ↓ Mitochondrial ROS generation at complex I, complex III, and reverse flow of electrons	[90]
	High-fat-diet-induced mice	Mung bean protein isolate (replacement of casein) in diet supplementation for 4 weeks	↓ Hepatic TG concentration ↑ Plasma TG concentrations ↓ The expression levels of hepatic lipogenic gene: Sterol regulatory element binding transcription factor 1, fatty acid synthase, stearoyl-coenzyme A desaturase 1, glucose-6-phosphate dehydrogenase 2, glucose-6-phosphate dehydrogenase X-linked	[91]
	Effect-ethanol induced hepatotoxicity in mice	Vitexin and isovitexin (15 and 13 mg/kg) for 14 days	↓ Serum ALT, AST ↑ SOD activity ↓ MDA activity ↓ Liver injury	[92]
	Effect-ethanol induced hepatotoxicity in mice	Aqueous extracts of germinated and fermented mung beans (200 and 1000 mg/kg) for 7 and 14 days	↓ Serum ALT, AST, TG, and cholesterol ↑ SOD, FRAP levels in liver ↓ MDA, NO levels in liver ↓ Hepatocyte damage	[52]
	Acetaminophen-induced acute hepatotoxicity model in rats	Aqueous extracts of the mung bean (100, 500, and 1000 mg/kg) for a single oral administration	↓ Serum glutamate-oxalate-transaminase, glutamate-pyruvate-transaminase ↓ Liver damaged by acetaminophen	[93]
Antihypertensive	Spontaneously hypertensive rats	Mung bean protein isolate hydrolysates (600 mg/kg) for a single oral administration	↓ SBP, heart rate	[94]
	Spontaneously hypertensive rats	Aqueous extracts and hydrolysates of mung bean sprouts (600 mg peptide/kg) for 8 weeks	↓ SBD, plasma ACE activity	[95]
Anticancer properties	Breast cancer cells 4T1 injected mice	Aqueous extract of fermented mung bean (200 and 1000 mg/kg) for 30 days	↓ Tumor formation ↑ Serum anticancer cytokine levels, spleen T cell populations, splenocyte cytotoxicity ↑ IL-2 and IFN-γ ↓ SOD and NO levels in the liver ↓ The lipid peroxidation ↓ Mitotic division in the tumors	[96]
Immunomodulatory activity	Cyclophosphamide induced-immunosuppressed model	Verbascose from mung bean (30, 90 and 270 mg/kg) for 8 days	↑ Spleen and thymus indices ↑ Swelling rate of earlap in the delayed type of hypersensitivity reaction ↑ Serum hemolysin ↑ Lysozyme in Serum and Spleen	[73]
	Arachidonic acid-induced ear edema in mice	Aqueous extract of untreated, germinated, and fermented mung bean (200 and 1000 mg/kg) for single oral administration	Exhibited the edema inhibition effect	[22]
	An animal model of sepsis induced by cecal ligation and puncture	Aqueous extract of mung bean seed coat (0.2 mL/mouse, containing 1.0 mg lyophilized extract) for 2 weeks	↑ Survival rates	[76]
	Complete Freund’s adjuvant-induced arthritis in rats	Ethanolic extract of mung bean (250 and 500 μg/mL) for 21 days	↑ Body weight, percentage inhibition of paw edema, pain threshold ↓ Serum TNF-α and IL-1β, IL-6 and IL-10 levels ↓ Liver lysosomal enzyme levels (*N*-acetyl-β-d-glusoaminidase, cathepsin-D, glucuronidase) ↓ MDA levels, myeloperoxidase activity ↑ Glutathione level	[97]
	Diabetic KK-Ay mice	Ethanolic extract of mung bean (1 g/kg) for 4 weeks	↓ Epididymal and perirenal fat weights ↑ Plasma TG and TC levels ↓ Plasma IL-6 levels, ↓ Intramuscular TNF-α and MCP-1 levels ↓ Intramuscular TG and TC levels ↓ The gene expression levels of p-AMPK, ACC, and PGC1α, p-ERK1/2, PPARγ, C/EBPα, and p-p38 in intramuscular	[67]

Note: ↑ and ↓ signs represent increase and decrease, respectively, after supplementation of the mung bean or its active compounds. Triglyceride (TG); total cholesterol (TC); non-high-density lipoprotein cholesterol (non-HDL-C); high-density lipoprotein cholesterol (HDL-C); low-density lipoprotein (LDL); free fatty acids (FFA); homeostasis model assessment of insulin resistance (HOMA-IR); superoxide dismutase (SOD); catalase (CAT); glutathione peroxidase (GSH-Px); cholesterol-7α-hydroxylase (CYP7A1); alanine aminotransferase (ALT); alkaline phosphatase (ALP); malondialdehyde (MDA); ferric ion reducing antioxidant power (FRAP); interleukin-10 (IL-10); (lipolysis marker) phosphorylated 5’ adenosine monophosphate-activated protein kinase (p-AMPK); acetyl-CoA carboxylase (ACC); proliferator-activated receptor gamma coactivator-1 alpha (PGC-1α); phosphorylated extracellular signal-regulated protein kinases ½ (p-ERK1/2); peroxisome proliferator-activated receptor (PPARγ); phosphorylated p38 mitogen-activated protein kinase (p-p38); thiobarbituric acid reactive substances (TBARS); glutathione reductase (GR); glucose-6 phosphate dehydrogenase (G6PDH); reactive oxygen species (ROS). Systolic blood pressure (SBP); diastolic blood pressure (DBP).
